# Bilateral adrenal tumors presenting with Cushing syndrome in the setting of horseshoe kidney: surgical and urological considerations (case report)

**DOI:** 10.11604/pamj.2025.52.48.49412

**Published:** 2025-09-30

**Authors:** Aulia Rahman Putra, Kurnia Penta Seputra, Taufiq Nur Budaya, Haryo Nindito Wicaksono

**Affiliations:** 1 Department of Urology, Faculty of Medicine, Universitas Brawijaya, Saiful Anwar General Hospital, Malang, Indonesia

**Keywords:** Cushing syndrome, adrenal tumor, horseshoe kidney, adrenalectomy, case report

## Abstract

Simultaneous bilateral adrenal masses are less common in patients with Cushing syndrome (CS). This case report focuses on the clinical features, diagnostic workup, and treatment of a patient with bilateral adrenal tumors causing Cushing syndrome, with a horseshoe kidney. We report a case of bilateral adrenal tumors presenting with Cushing syndrome with a horseshoe kidney. Adrenalectomy was undertaken to manage the excess cortisol. Histopathological evaluation confirmed the diagnosis of adrenal cortical carcinoma (ACC). The preoperative cortisol level was 1750 nmol/L, which dropped significantly to 476 nmol/L following the treatment, showing clinical improvement, with a gradual regression of Cushing syndrome symptoms. A three-month postoperative evaluation revealed cortisol levels of 143 nmol/L. Surgical resection of these tumors has the potential to significantly improve clinical outcomes and alleviate the manifestations of Cushing syndrome.

## Introduction

Simultaneous bilateral adrenal masses are markedly less common [1]. Bilateral adrenal masses are more frequently associated with malignancy and tumor susceptibility syndromes [2]. They are also more likely to be associated with Cushing syndrome. It is associated with increased morbidity and mortality due to hypertension, diabetes, coagulopathy, cardiovascular disease, and fractures [2]. In certain cases, surgical treatment may be necessary. This case report presents a rare instance of CS caused by bilateral adrenal tumors, an uncommon source of hypercortisolism with horseshoe kidney.

## Patient and observation

**Patient information:** a 58-year-old female patient, hypertensive and type 2 diabetes.

**Clinical findings:** on physical examination, she presented classic cushingoid features, including a moon face, fine facial hair growth, and purplish abdominal striae. There was evidence of systemic fluid retention with bilateral lower limb edema, and pressure ulcers were noted on the gluteal region. Costovertebral angle percussion elicited pain, while the rest of the systemic examination was unremarkable.

**Figure 1:**
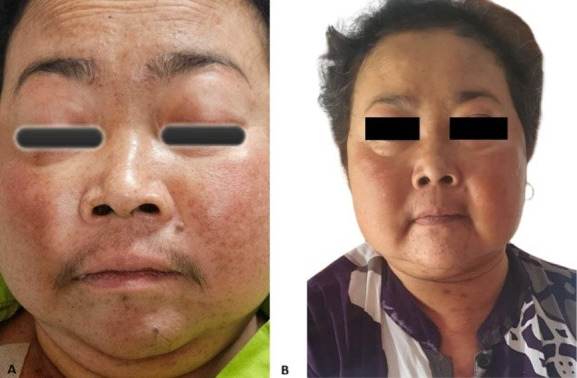
A) patient with progressive facial swelling and fine hair growth over her face; B) after surgery, the facial hair disappeared, and the facial swelling disappeared

**Timeline of current episode:** February 2015: the patient was diagnosed with type 2 diabetes mellitus, managed with insulin, and hypertension, which was not well-controlled on medication. February 2024: onset of progressive bilateral flank pain, more severe on the left, described as dull and non-radiating, worsened with activity and relieved with rest; the patient self-medicated with paracetamol. April 2024: progressive facial swelling developed, accompanied by the appearance of fine facial hair (hirsutism). In the following months, generalized weakness was noted, along with painless ulcers over the buttocks. June 2024: worsening of flank pain occurred, particularly on the left side. July 2024: the patient experienced persistent bilateral flank pain (left greater than right), facial swelling, hirsutism, generalized weakness, and buttock ulcers (Figure 1, Table 1).

**Table 1:** timeline of the current episode

February 2015	Diagnosed with type 2 diabetes mellitus → managed with insulin. Diagnosed with hypertension, not well-controlled on medication.
February 2024	Onset of progressive bilateral flank pain (more severe on left) Dull, non-radiating, worsened with activity, relieved with rest. Self-medicated with paracetamol.
April 2024	Developed progressive facial swelling. Appearance of fine facial hair (hirsutism).
Following months	Experienced generalized weakness. Developed painless ulcers over the buttocks.
June 2024	Worsening of flank pain, especially on the left side.
July 2024	Persistent bilateral flank pain (L > R). Facial swelling, hirsutism, generalized weakness, and buttock ulcers are present.

**Figure 2:**
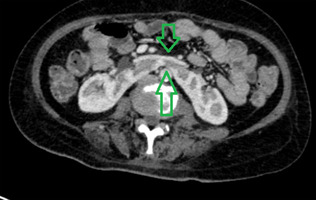
computed tomography scan of coronal section: green arrows indicate the area of fused left and right kidney forming a horseshoe kidney formation retroperitoneally near the lumbar spine

**Figure 3:**
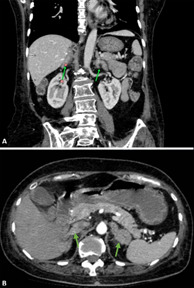
(A,B) computed tomography scan of coronal and transverse section: green arrows indicate both left and right enlarged adrenal glands

**Diagnostic assessment:** laboratory and radiological assessments were done. Laboratory work-up revealed an elevated HbA1c (6.1%), hypercortisolism with significantly increased cortisol levels (811 nmol/L), hypokalemia (2.15 mEq/L), hypoalbuminemia (2.94 g/dL), and metabolic alkalosis. Radiological studies provided further diagnostic insight. An initial abdominal ultrasound was unremarkable, but contrast-enhanced CT scanning revealed bilateral adrenal gland enlargement with post-contrast enhancement and surrounding fat stranding, suggestive of adrenal hyperplasia (right adrenal: 1.7 x 1.7 x 4.8 cm; left adrenal: 1.4 x 3.2 x 2.5 cm). An incidental horseshoe kidney was also identified, as shown in Figure 2, Figure 3. CT angiography showed no evidence of vascular anomalies or renal artery stenosis, and an MRI of the brain ruled out pituitary involvement. The preoperative cortisol level was 1750 nmol/L.

**Diagnosis:** patient was diagnosed with CS due to bilateral adrenal tumors (cT1N0M0) with an incidental horseshoe kidney. Pathology Evaluation confirmed Adrenal Cortical Carcinoma (ACC).

**Figure 4:**
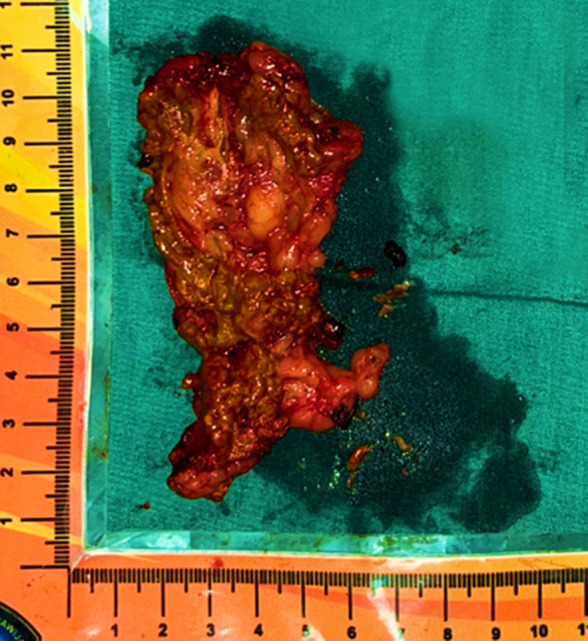
post-resection left adrenal gland with nodules inside

**Therapeutic interventions:** left adrenalectomy was performed as definitive management for cortisol excess and sent to pathology for examination. The Post-resection left adrenal gland with nodules inside can be seen in Figure 4. The decision to perform a left unilateral adrenalectomy was based on the patient´s more pronounced pain on the left flank and the clinical belief that the left adrenal gland was the primary contributor to cortisol excess. By removing only one adrenal gland, the patient could also retain adequate natural cortisol production, minimizing the need for lifelong steroid therapy and lowering the risk of adrenal insufficiency.

**Follow-up and outcome of interventions:** the patient demonstrated favorable postoperative outcomes, accompanied by noticeable clinical improvement. There was a gradual regression of Cushing syndrome symptoms, including improvements in physical appearance and overall functional status. A three-month postoperative evaluation revealed cortisol levels of 143 nmol/L.

**Patient perspective:** “I felt unwell for months, but after surgery, I was relieved to see my symptoms improving. The lower cortisol levels gave me hope, and despite the cancer diagnosis, I remain grateful and optimistic about my recovery.”

**Informed consent:** this study was approved by the Health Research Ethics Committee of Dr. Saiful Anwar General Hospital with ethical clearance number 400/018/CR/102.7/2025. The patient was informed in detail about the study objectives, procedures, potential benefits, and they signed the informed consent form after fully understanding the provided information.

## Discussion

Our patient displayed both clinical and biochemical evidence of hypercortisolism and met the criteria for CS diagnosis. Adrenal cortical carcinoma (ACC) is a rare tumor of the adrenal cortex. The incidence of ACC follows a bimodal age distribution, with a plateau between 40 and 50 years of age. The incidence is more frequent in women (sex ratio 1.5). Adrenal cortical carcinoma (ACC) constitutes about 8-11% of adrenal tumors in clinical and surgical series [3]. The statement is consistent with the patient's gender and age in this case report. Patients with CS present with obesity or weight gain (95% of cases), facial plethora (90%), moon face (90%), thinning of skin (85%), menstrual irregularity (80%), hypertension (75%), hirsutism (75%), and others [4]. Our patient presented with typical symptoms. Notably, her chief complaint was bilateral flank pain rather than symptoms directly attributable to cortisol excess. This atypical pain may be secondary to compression or irritation of adjacent structures by enlarged adrenal tissue or could reflect altered anatomical relationships due to an incidental congenital anomaly.

The incidental discovery of a horseshoe kidney adds complexity to the case. Although often asymptomatic, horseshoe kidneys can occasionally cause symptoms, such as flank pain, due to altered drainage or mass effects. Similar cases in the literature highlight its rarity and the diagnostic challenges it presents. Araujo-Castro and Marazuela analyzed bilateral adrenal cortical disease with complex anatomical variants, emphasizing the need for comprehensive evaluation. The coexistence of bilateral adrenal tumors and a horseshoe kidney has significant diagnostic and therapeutic implications, requiring meticulous preoperative planning to preserve renal function and optimize the surgical approach [5]. Recent treatment strategies for Cushing´s disease focus on correcting cortisol excess, restoring the underlying hormonal axis, and managing subsequent sequelae. Treatment options vary widely according to the underlying etiology and include unilateral or bilateral adrenalectomy. Reibetanz *et al*. [6] observed that among 83 patients undergoing adrenalectomy for hypercortisolism, the study reported significantly higher severe complication rates (33% vs. 0%, p<0.001), and delayed recovery (median 79.2% vs. 10.2%, p<0.001) following bilateral adrenalectomy compared to unilateral adrenalectomy, with a trend towards increased postoperative mortality in the bilateral group (8.3% vs. 0%, p=0.081).

**Figure 5:**
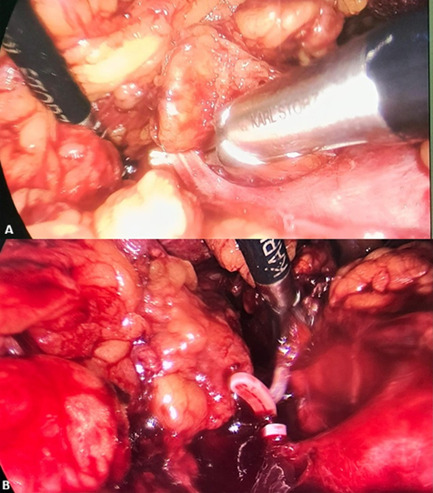
(A,B) identification and clamping of the suprarenal vessels; mass resection using a laparoscopic approach

The removal of a solitary adrenal gland is sufficient to ensure adequate endogenous cortisol production, thereby circumventing the necessity for lifelong glucocorticoid replacement and mitigating risks such as adrenal insufficiency. Data demonstrate that in cases of bilateral disease, preserving at least a portion of one gland is associated with a success rate of over 85-90% of patients maintaining steroid independence post-surgery. Bilateral adrenalectomy often results in lifelong adrenal insufficiency, necessitating permanent steroid replacement therapy. This condition is associated with an increased risk of morbidity and a diminished quality of life [7,8]. A left adrenalectomy began with incisions similar to those used on the right side, followed by division of the phrenocolic ligament and medial mobilization of the descending colon. In cases of large tumors, the inferior mesenteric vein was ligated and the ligament of Treitz divided. The posterior peritoneum was then incised along the inferior border of the pancreas to expose and open Gerot´s fascia. During adrenalectomy, the inferior adrenal vein was ligated first, followed sequentially by the inferior adrenal artery and the central adrenal vein. Finally, the superior adrenal vessels, along with the medial arterial and venous branches, were ligated [9] (Figure 5). Follow-up for patients with adrenocortical carcinoma is typically scheduled 2 to 6 weeks after surgery to evaluate postoperative hormone levels. Subsequent follow-ups consist of clinical and radiological assessments every 3 months during the first 2 to 3 years, and every 6 months thereafter for up to 5 years to monitor for recurrence [10].

**Limitation:** adrenocorticotropic hormone (ACTH) measurement was not available at the time of diagnosis, representing a limitation in definitively classifying the CS as ACTH-independent, although imaging and clinical features strongly supported this etiology.

## Conclusion

A comprehensive evaluation of patients with CS should include consideration of adrenal tumors as a potential underlying cause. Surgical resection of these tumors has the potential to significantly improve clinical outcomes and alleviate the manifestations of CS. Further studies are needed to determine whether this coexistence is coincidental or reflects a shared developmental pathway, which would guide management in complex cases.
